# Quantitative DNA Methylation Analysis of DLGAP2 Gene using Pyrosequencing in Schizophrenia with Tardive Dyskinesia: A Linear Mixed Model Approach

**DOI:** 10.1038/s41598-018-35718-4

**Published:** 2018-11-30

**Authors:** Yanli Li, Kesheng Wang, Ping Zhang, Junchao Huang, Huimei An, Nianyang Wang, Fu De Yang, Zhiren Wang, Shuping Tan, Song Chen, Yunlong Tan

**Affiliations:** 1 Beijing HuiLongGuan Hospital, Peking University HuiLongGuan Clinical Medical School, Beijing, 100096 China; 2 Department of Biostatistics and Epidemiology, College of Public Health, East Tennessee State University, Johnson City, TN 37614 USA; 3 Division of Biostatistics, Department of Preventive Medicine, Feinberg School of Medicine, Northwestern University, Chicago, IL 60611 USA

## Abstract

Tardive dyskinesia (TD) is a side effect of antipsychotic medications used to treat schizophrenia (SCZ) and other mental health disorders. No study has previously used pyrosequencing to quantify DNA methylation levels of the *DLGAP2* gene; while the quantitative methylation levels among CpG sites within a gene may be correlated. To deal with the correlated measures among three CpG sites within the *DLGAP2* gene, this study analyzed DNA methylation levels of the *DLGAP2* gene using a linear mixed model (LMM) in a Chinese sample consisting of 35 SCZ patients with TD, 35 SCZ without TD (NTD) and 34 healthy controls (HCs) collected in Beijing, China. The initial analysis using the non-parametric Kruskal-Wallis test revealed that three groups (TD, NTD and HC) had significant differences in DNA methylation level for CpG site 2 (p = 0.0119). Furthermore, the average methylation levels among the three CpG sites showed strong correlations (all p values < 0.0001). In addition, using the LMM, three groups had significant differences in methylation level (p = 0.0027); while TD, NTD and TD + NTD groups showed higher average methylation levels than the HC group (p = 0.0024, 0.0151, and 0.0007, respectively). In conclusion, the LMM can accommodate a covariance structure. The findings of this study provide first evidence of DNA methylation levels in *DLGAP2* associated with SCZ with TD in Chinese population. However, TD just showed borderline significant differences to NTD in this study.

## Introduction

Schizophrenia (SCZ) has a prevalence of approximately 1% worldwide and represents a major public health concern. SCZ is known to be a multifactorial disorder, by the contribution of multiple susceptibility genes, which may interact with epigenetic processes and environmental factors, with a heritability between 60 and 80%^1–6^. Tardive dyskinesia (TD) is a side effect of antipsychotic medications which are used to treat SCZ and other mental health disorders. The prevalence of TD in SCZ can range from 20% to 50%^7,8^. It has been proposed that the occurrence of TD may be the result of the interactions between genetics, environment, and epigenetics^8–11^.

Epigenetic changes affect gene expression and function by mechanisms other than those from changes in the DNA sequence; whereas DNA methylation is an important epigenetic modification and involves the addition of a methyl group at the 5th carbon of cytosines preceding guanines (CpG dinucleotides). DNA methylation has been shown to regulate gene expression when implicated in SCZ^12–16^ and TD^17^. Pyrosequencing offers a robust, versatile platform yielding rapid quantitative analysis of DNA methylation levels and providing information on the methylation status of single CpG sites^18–20^.

The *DLGAP2* gene (also known as *DAP2*, *SAPAP2*, *C8orf68*, and *ERICH1*-*AS1*) is located at 8p23.3 and is highly expressed in the striatum and may play a role in the molecular organization of synapses and in neuronal cell signaling^21,22^. Recently, *DLGAP2* was found to be associated with SCZ^23,24^ and several single nucleotide variations in *DLGAP2* have been reported in SCZ patient cohorts^25,26^. More recently, a review has focused on the direct and indirect role of the DLGAP family on SCZ as well as other brain diseases^27^. Regarding methylation, the *DLGAP2* gene revealed differences in methylation status when SCZ patients with healthy controls^13,14^. However, no study has used pyrosequencing to quantify DNA methylation levels of the *DLGAP2* gene; in addition, no study has examined the methylation of *DLGAP2* gene in TD. Furthermore, the DNA methylation levels using pyrosequencing among CpG sites within a gene may be correlated^28^. Mixed models (also known as multilevel models or hierarchical models) including both fixed effects and random effects have been developed to deal with correlated data^29–33^. However, few studies have been found to use mixed models in methylation analysis^34–37^. In addition, no study has been found to study DNA methylation of *DLGAP2* gene in SCZ or TD within the Chinese sample. Therefore, this study sought to quantify DNA methylation levels of *DLGAP2* gene in SCZ with or without TD using pyrosequencing and to deal with the possible correlations among 3 CpG sites within the *DLGAP2* gene using a linear mixed model (LMM) in a Chinese population.

## Results

### Descriptive statistics

The demographic and DNA methylation levels among the three groups (TD, NTD and HC) were summarized in Table 1. There were no statistical significances in age (p = 0.993) and sex composition (p =  0.983) among the three groups; whereas there were significant differences in the DNA methylation levels in site 2 and the average of three CpG sites among three groups using GLM (p = 0.0307 and 0.0465, respectively).

**Table 1:** Descriptive characteristics of patients and controls.

Variable	TD group (n = 35)	NTD group (n = 35)	HC group (n = 34)	χ^2^/F value	p value
Male/Female	20/15	20/15	19/15	0.0148	0.993
Age (year)	45.1 ± 12.2	44.7 ± 11.2	44.4 ± 11.6	0.02	0.983
DLGAP2 Site 1	91.9 ± 2.6	91.9 ± 2.0	90.4 ± 7.0	1.29	0.280
DLGAP2 Site 2	74.7 ± 4.6	73.4 ± 5.0	70.8 ± 8.4	3.61	0.0307
DLGAP2 Site 3	94.2 ± 1.3	94.3 ± 1.1	93.4 ± 4.0	1.81	0.169
DLGAP2 Average	86.9 ± 1.6	86.5 ± 1.8	84.9 ± 6.0	3.16	0.0465

Abbreviations: TD = schizophrenia patients with tardive dyskinesia (TD), NTD = schizophrenia patients without TD, HC = healthy controls, χ^2^ value is based on the chi-square test, F value is based on the generalized linear model.

### Kruskal-Wallis test

**Figure 1:**
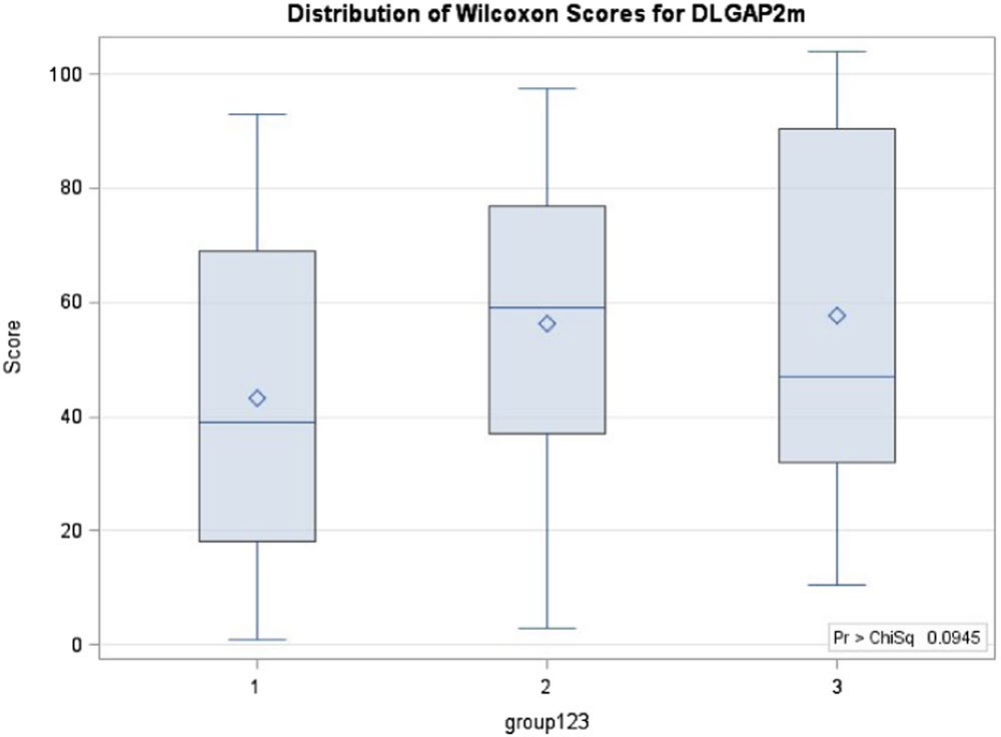
Wilcoxon scores for the average methylation of 3 sites for DLGAP2 gene when comparison of TD vs NTD vs HC. 1 refers to HC, 2 refers to NTD, 3 refers to TD.

**Figure 2:**
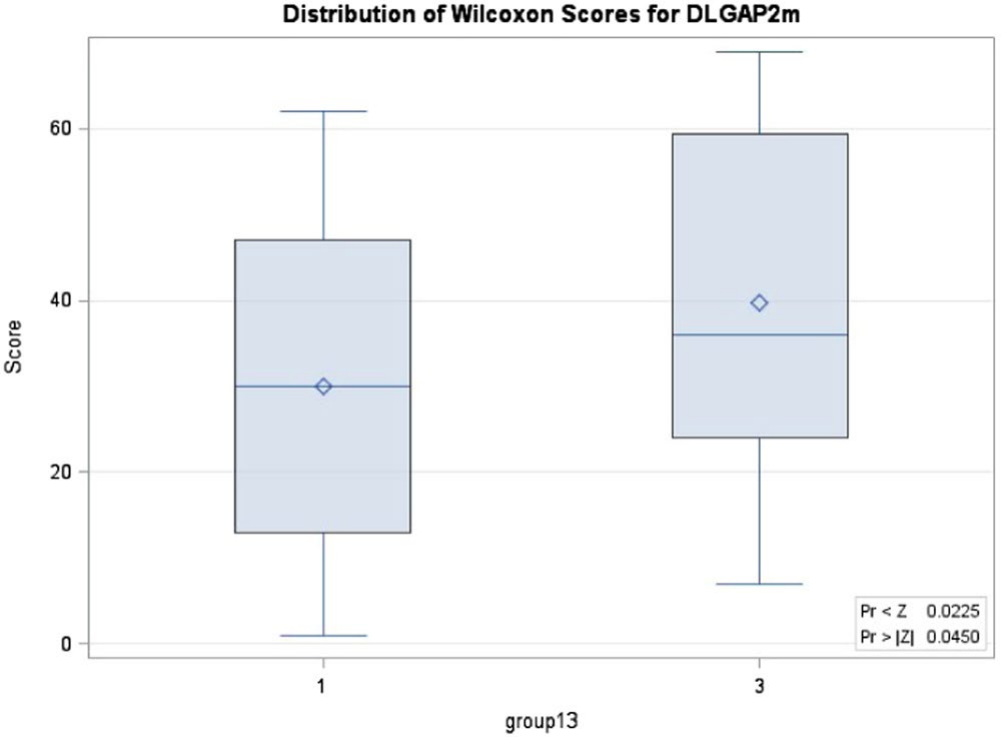
Wilcoxon scores for the average methylation of 3 sites for DLGAP2 gene when comparison of TD with HC. 1 refers to HC, 3 refers to TD. P value of 0.0225 is based on one-side test and 0.0450 is based on two-side test.

**Figure 3:**
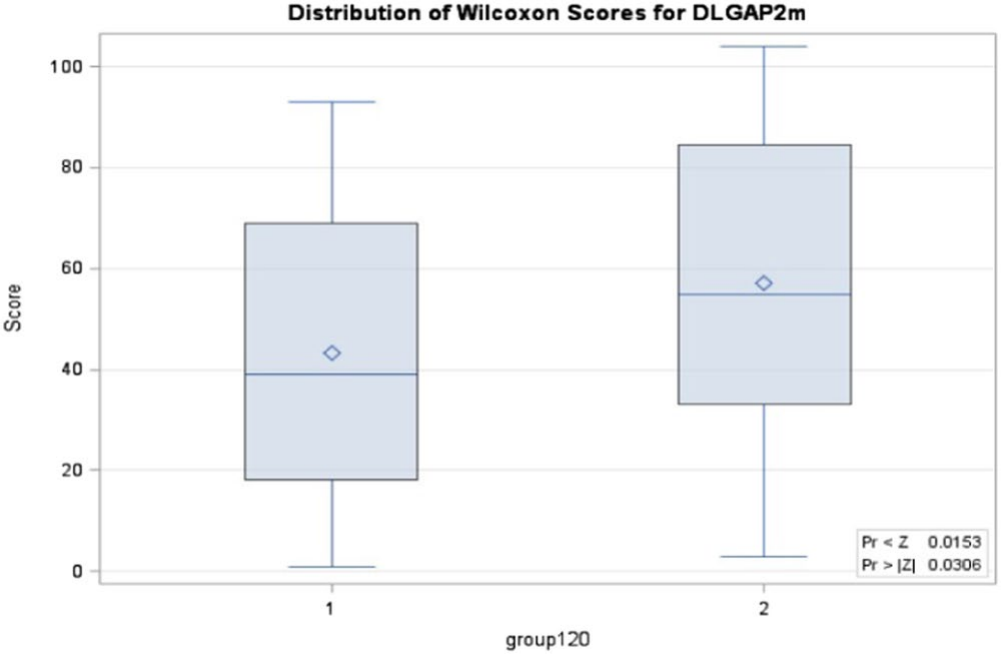
Wilcoxon scores for the average methylation of 3 sites for DLGAP2 gene when comparison of TD + NTD with HC. 1 refers to HC, 2 refers to TD + NTD. P value of 0.0153 is based on one-side test and 0.0306 is based on two-side test.

The Kruskal-Wallis test revealed that the three groups had significant differences in DNA methylation level for CpG site 2 (p = 0.0119) and borderline differences for the average DNA methylation level of 3 CpG sites (p = 0.0945) (Table 2 and Fig. 1). Furthermore, the Wilcoxon test showed that one-sided p values when comparing the methylation level in site 2 of TD, NTD and ND + NTD with HC group were 0.0038, 0.0252 and 0.0038, respectively; while TD also revealed borderline significance comparing with NTD (p = 0.0559). For CpG site 3, the comparison of TD and ND + NTD groups with the HC group were significant (one-sided p = 0.0161 and 0.0195, respectively). In addition, comparing the average methylation levels of TD, NTD and ND + NTD groups with the HC group, the one-sided p values were 0.0225, 0.0409 and 0.0153, respectively (Table 2, Figs 2 and 3).

**Table 2:** Kruskal-Wallis test of methylation levels in DLGAP2 gene.

Test	Group	Scores/χ^2^/p value	Site 1	Site 2	Site 3	Average
**KW test**						
	TD	mean score	57.8	62.7	58.8	57.7
	NTD		53.0	53.3	54.7	56.2
	HC		46.6	41.2	43.7	43.3
	TD vs NTD vs HC	χ^2^	2.39	8.86	4.59	4.72
		p value	0.302	0.0119	0.101	0.0945
**Wilcoxon test**						
	TD vs HC	p value (one-sided)	0.0716	0.0038	0.0161	0.0225
		p value (two-sided)	0.143	0.0076	0.0322	0.0450
	NTD vs HC	p value (one-sided)	0.17	0.0252	0.0758	0.0409
		p value (two-sided)	0.34	0.0504	0.152	0.0818
	TD + NTD vs HC	p value (one-sided)	0.0828	0.0038	0.0195	0.0153
		p value (two-sided)	0.1626	0.0076	0.0389	0.0306
	TD vs NTD	p value (one-sided)	0.229	0.0559	0.307	0.428
		p value (two-sided)	0.469	0.110	0.614	0.855

Abbreviations: TD = schizophrenia patients with tardive dyskinesia (TD), NTD = schizophrenia patients without TD, HC = healthy controls, KW test refers to Kruskal-Wallis test for comparison of 3 groups, mean score is based on the rank, χ^2^ is based on the chi-square test for the KW test, one sided and two-sided p values are based on the Wilcoxon test for comparison of 2 groups.

### Correlation analysis

Table 3 shows that there were significant correlations among the DNA methylation levels of 3 CpG sites and the average of 3 CpG sites (all p values < 0.0001).

**Table 3:** Correlation analysis.

Variable	DLGAP2 Site 1	DLGAP2 Site 2	DLGAP2 Site 3	DLGAP2 Average	Age
DLGAP2 Site 1	1.000	0.480****	0.896****	0.858****	−0.161
DLGAP2 Site 2		1.000	0.495****	0.855****	0.207*
DLGAP2 Site 3			1.000	0.848****	−0.173
DLGAP2 Average				1.000	0.0142

Abbreviations: *Refers to p < 5% in Persona correlation analysis, ****refers to p < 0.0001 in Persona correlation analysis.

### Linear mixed model analysis

Random effect test results are presented in Table 4. Using the default model (the covariance structure is variance components) in the LMM^30^, the three CpG sites showed significant differences for all comparisons (all p values of random effects were <0.0001) in Table 4. Furthermore, the 3 groups had significant difference in DNA methylation level (p = 0.0027); while CpG sites 1 and 2 showed significant lower DNA methylation levels than in CpG site 3 (*t* test in Table 5). In addition, TD, NTD and ND + NTD groups showed significant differences from the HC group (p = 0.0042, 0.0151, and 0.0007, respectively) in Table 5.

**Table 4:** Random effects using linear mixed models.

Cov Parm	Estimate	SE	Z value	p value
Site for TD vs. NTD vs HC	0.00309	0.000248	12.49	<0.0001
Site for TD vs HC	0.00387	0.000381	10.17	<0.0001
Site for NTD vs HC	0.00375	0.000369	10.17	<0.0001
Site for TD + NTD vs HC	0.00310	0.000248	12.49	<0.0001
Site for TD vs NTD	0.00166	0.000162	10.25	<0.0001

Abbreviations: TD = schizophrenia patients with tardive dyskinesia (TD), NTD = schizophrenia patients without TD, HC = healthy controls, Cov Parm = Covariance Parameter, SE = Standard Error, Z value and p value are based on the Z test for the random effect in the linear mixed models.

**Table 5:** Fixed effects using linear mixed models.

Group	Num DF	Den DF	F/t value	p value
TD vs. NTD vs HC	2	101	6.26	0.0027
Site	2	206	871.4	<0.0001
Site 1 vs 3			−6.20	<0.0001
Site 2 vs 3			−38.5	<0.0001
TD vs HC	1	67	8.78	0.0042
Site	2	136	457.74	<0.0001
Site 1 vs 3			−4.51	<0.0001
Site 2 vs 3			−28.16	<0.0001
NTD vs HC	1	67	6.22	0.0151
Site	2	136	501.15	<0.0001
Site 1 vs 3			−4.73	<0.0001
Site 2 vs 3			−29.48	<0.0001
TD + NTD vs HC	1	102	12.18	0.0007
Site	2	206	870.49	<0.0001
Site 1 vs 3			−6.20	<0.0001
Site 2 vs 3			−38.83	<0.0001
TD vs NTD	1	68	0.62	0.436
Site	2	138	1050.4	<0.0001
Site 1 vs 3			−6.74	<0.0001
Site 2 vs 3	2	138	−42.63	<0.0001

Abbreviations: TD = schizophrenia patients with tardive dyskinesia (TD), NTD = schizophrenia patients without TD, HC = healthy controls, Num DF refers to the number of degrees of freedom in the model, Den DF refers to the number of degrees of freedom associated with the model errors, F/t value and p value are based on the linear mixed models.

## Discussion

In this study, we performed pyrosequencing analysis to determine *DLGAP2* gene promoter methylation levels among TD, NTD and HC groups. Both the non-parametric method and LMM revealed significant increases of DNA methylation in TD and NTD groups compared with healthy controls. However, TD showed only borderline significant differences to NTD in CpG site 2 using the Wilcoxon rank test. To our knowledge, this is the first study to compare the quantitative DNA methylation levels using pyrosequencing in *DLGAP2* gene of TD compared to NTD and healthy controls.

Wockner *et al*.^13^ performed a genome-wide DNA methylation analysis on post-mortem human brain tissue from 24 patients with SCZ and 24 unaffected controls using the Illumina Infinium HumanMethylation450 Bead Chip. They found that the M value = 0.324 (the log2 ratio of the intensities of methylated probe versus unmethylated probe) with p = 0.00035985 (adjusted p = 0.04194) for the *DLGAP2* gene as stated in Table S1^13^. Another study reported that *DLGAP2* showed a DNA methylation change of −1.13 with p = 4.59 × 10^−5^ in SCZ^14^. In the present study we confirmed that SCZ (NTD group) has significant increased DNA methylation levels compared to healthy controls using pyrosequencing. We further added that TD has significant increased DNA methylation levels compared to healthy controls using pyrosequencing. However, TD showed only borderline significant differences to NTD in CpG site 2 in the *DLGAP2* using the Wilcoxon rank test.

Previous studies have shown that *DLGAP2* may play a role in the molecular organization of synapses and in neuronal cell signaling^21^. Another study found *DLGAP2* is highly expressed in the striatum; however, *Dlgap2* is the only *Dlgap* that is not expressed in the cerebellum and in the thalamus^22^. Furthermore, genetic association studies have shown that polymorphism in *DLGAP2* has been associated with SCZ as well as other brain diseases^25–27^. However, no study has been found to quantify DNA methylation levels of the *DLGAP2* gene using pyrosequencing in NTD and to examine the methylation of *DLGAP2* gene in TD. Previous studies suggested that the occurrence of TD may be the result of the interactions between genetics, environment, and epigenetics^8–11^. Furthermore, it is proposed that antipsychotics are epigenetic modifiers with widespread effects on site-specific and global DNA methylation^38^. Recently, Zhang *et al*. (2018) reported the preliminary DNA methylation profiles in SCZ with TD by using methylated DNA immunoprecipitation coupled with next-generation sequencing in a case-control design in a Chinese sample^17^ and found that 161 genes were specific to TD group; however, the *DLGAP2* gene was not reported. The present study provided the first evidence of the DNA methylation levels of the *DLGAP2* gene associated with pathogenesis of TD.

Pyrosequencing allows the simultaneous analysis of several CpG sites up to 100 bp amplicon length and offers a robust, versatile platform yielding rapid quantitative results^18–20^. Previous studies have used the t-test or ANOVA^39–41^ or the non-parametric Kruskal-Wallis test^42–44^ to evaluate the differences of DNA methylation levels in single CpG site or the average of several sites among groups. However, the DNA methylation levels among CpG sites within a gene may be correlated^28^. Our present study also revealed that there were significant correlations among the methylation percentages of 3 CpG sites and the average of 3 CpG sites (p < 0.0001) (Table 3). Ignoring the correlation among sites may cause bias in the relationship. The LMM has been proposed to analyze correlated quantitative data^30,31,33^; however, only a few studies have used LMM in the analysis of DNA methylation levels^34–37^. In the present study, we used the LMM to deal with the correlated structures among 3 CpG sites within the *DLGAP2* gene and found that the differences in DNA methylation levels among 3 groups were stronger using LMM than using non-parametric Kruskal-Wallis test (Tables 2 and 5).

This study has several strengths. First, we performed the first quantitative analysis of DNA methylation levels of the *DLGAP2* gene in SCZ with TD using pyrosequencing. Second, the present study attempted to use LMM in analysis of correlated DNA methylation levels within a gene. However, this study also has some limitations. First, DNA methylation has tissue specificity; however, it is difficult to sample brain tissues for researches on the central nervous system diseases, the biological samples selected here were peripheral blood. Second, we studied limited sites of *DLGAP2* methylation (only 3 CpG sites). Third, the sample size is relatively small. We used PROC MIXED in SAS 9.4 to compute power for the three independent groups^45,46^. Based on our sample size of 104 individuals, the power to detect the difference among overall means for 3 CpG sites could reach 50%; while the power could reach 97% when we just considered CpG site 2. We considered complex designs including random and fixed effects in the LMMs (Tables 4 and 5), the power would then be higher^45^.

In conclusion the LMM can be used to deal with complex relationships in DNA methylation levels among CpG sites. This study showed increased DNA methylation levels of the *DLGAP2* gene in both TD and NTD patients compared to control individuals. However, TD showed only borderline significant differences to NTD in CpG site 2 using the Wilcoxon rank test. Further studies will be essential to examine age, gender and racial effects using LMM and large sample. In addition, further functional analysis of methylation level of these 3 CpG sites of DLGAP2 gene may help to better understand the mechanisms of this gene on the development of SCZ and TD.

## Methods

### The Chinese sample

This methylation study consists of 35 SCZ patients with TD, 35 SCZ non-TD (NTD) patients and 35 healthy controls (HCs). 70 patients with SCZ were recruited from December 2016 to August 2017 in Beijing HuiLongGuan Hospital (Beijing, China). SCZ was diagnosed using DSM-IV. Clinical diagnoses of TD were confirmed by two highly experienced psychiatrists according to the criteria of Schooler and Kane^47^. Inclusion criteria for TD group include having ages between 18 and 40 years old; with Abnormal Involuntary Movement Scale (AIMS) scored larger than 3 in at least one part or at least 2 in two or more parts. The same criteria were used for NTD group except that AIMS  = 0. Patients with any of the following situation were excluded: (1) severe physical or organic encephalopathy; (2) drug or alcohol abuse history (except tobacco); (3) pregnant or lactating women; (4) administration of neurotrophic agents or free radical metabolism drugs within 12 weeks prior to participation; (5) meeting other mental illness diagnosis of DSM-IV Axis I. 35 healthy controls demographically matched for age, sex, and education were enrolled at the same period from the local community.

### Ethics approval and consent to participate

All the individuals were of the Han Chinese ethnicity and live in Beijing. All subjects in the methylation study gave informed consent and were given written instructions to fast overnight before the venous blood sampling. Ethical approval for the methylation study was approved by the Ethics Review Board of Beijing HuiLongGuan Hospital. All methods were performed in accordance with the relevant guidelines and regulations.

### DNA extraction and pyrosequencing

Genomic DNA was extracted using a standard genomic DNA sample kit (Illumina) with the concentration and purity detected by NanoDrop spectrophotometer (NanoDrop Technologies, USA), and integrity tested using 1% agarose gel electrophoresis. Pyrosequencing (Pyro-Seq) was used to quantify one or more methylation sites. Pyrosequencing was performed for all the study samples on a PyroMark Q96 ID using Pyro Mark Gold reagents (Qiagen). Primers for *DLGAP2*, targeting 3 CpGs in the gene promoter, were generated according to Pyro Mark Assay Design software version 2.0 (Qiagen). Primer sequences are listed in Figure S1. Pyromark Q96 ID version 1.0.9 software was used to generate and automatically analyze pyrograms resulting from sequencing onto the PyroMark Q96 ID system. This study includes 3 CpG sites in the *DLGAP2* gene. Quantitative methylation results were considered both as percentage of individual CpG sites and as average of the methylation percentage of the 3 investigated CpGs (Figure S2). After quality control, our final sample size consisted of 35 SCZ with TD, 35 SCZ without TD and 34 controls.

### Descriptive statistics

The chi-square (χ^2^) test was used to analyze the gender differences across TD, NTD and HC groups. The age and the differences in the percentage of methylation among TD, NTD and HCs for each CpG site and the average of the methylation percentage of the 3 investigated CpGs were compared using the F test in a generalized linear model (GLM).

### Non-parametric Kruskal-Wallis test

The non-parametric Kruskal-Wallis test was initially used to compare the ranks of the DNA methylation levels for each CpG site and the average of the 3 CpGs among TD, NTD and HC groups. Then the Wilcoxon rank-sum test was used to compare the observations from any of two groups.

### Correlation analysis

Pearson’s correlation analysis was performed to test for correlation in methylation percentages among the 3 CpG sites, the average of the 3 CpG sites and age.

### Linear mixed models (LMM)

We considered the possible correlations among CpG sites within a gene, then the linear mixed model (LMM) was used^30,31,33,48^. The LMM included group as the fixed effect and sites as the random effect and was used to examine the DNA methylation differences among groups (1). The PROC MIXED procedure in SAS 9.4 was used to deal with the correlated measures.

1 $${{\rm{Y}}}_{{\bf{it}}}={\mu }_{t}+{\beta }_{i}{x}_{it}+\gamma {z}_{i}+{\alpha }_{i}+{\varepsilon }_{it}\,i=1,\ldots ,\,{\rm{n}};t=1,\ldots ,{\rm{T}}$$

where, Y_*it*_ is the value of the outcome for individual _*i*_ at site _*t*_, *μ*_*t*_ is an intercept varying with site, *x*_*it*_ is a vector of site-varying variables, *z*_*i*_ is a vector of site-invariant variables such as gender and race; *α*_*i*_ denotes the random effects with each having a normal distribution with a mean of 0 and constant variance, and *β* are fixed effects. *ε*_*ij*_ is a random distribution term. *i* = 1, …, I_j_ is level-1 individual *i* indicator, and *t* = 1, 2, 3 is the level-2 indicator such as 3 sites of CpG. Z test was used to examine the random effect; while F test was used to compare DNA methylation levels among groups and t test was used to compare the site effect. Before conducting analysis using LMMs, we performed log transformation of the DNA methylation levels.

All above analyses were performed with SAS version 9.4 (SAS Institute, Cary, NC, USA).

## Electronic supplementary material

Figures S1 and S2

## Data Availability

The methylation data are available from the corresponding authors on reasonable request.
