# Supplementary material for: Quantitative DNA Methylation Analysis of DLGAP2 Gene using Pyrosequencing in Schizophrenia with Tardive Dyskinesia: A Linear Mixed Model Approach
Source: Sci Rep. 2018 Nov 30;8:17466. doi: 10.1038/s41598-018-35718-4 (PMC6269460; doi:10.1038/s41598-018-35718-4)
Supplement: Supplementary file 1 — Figures S1 and S2 [file 41598_2018_35718_MOESM1_ESM.pdf]

Quantitative DNA Methylation Analysis of DLGAP2 Gene using Pyrosequencing in Schizophrenia with Tardive Dyskinesia: A Linear Mixed Model Approach

Yanli Li[1,†], Kesheng Wang[1,2*,†], Ping Zhang[1], Junchao Huang[1], Huimei An[1], Nianyang Wang[3], Fu De Yang[1], Zhiren Wang[1], Shuping Tan[1], Song Chen[1], Yunlong Tan[1*]

GTTAAAGAAAACTACAGCCAAAAAGTGTTTGCTTTTCAATATAAGATGAT
GGAAATGGGTAGAGTTCTAAAGGATGACCGTCCATTATCCACTGCAACTG
AGTCCTAGATTTGTTGCTAACTGACGTTCTACACAGCCTTTCTTCACTCGGAAATAGACGAGTGA
AAGTATTTCGACTTCTCATTAGGAAGGAACAGTGGctatcatgtttgaataattatgcatctgttttttta
aacctcagtctggcaatactccatcgcgatgaatcttcactactcagcctgcctggggaggctt

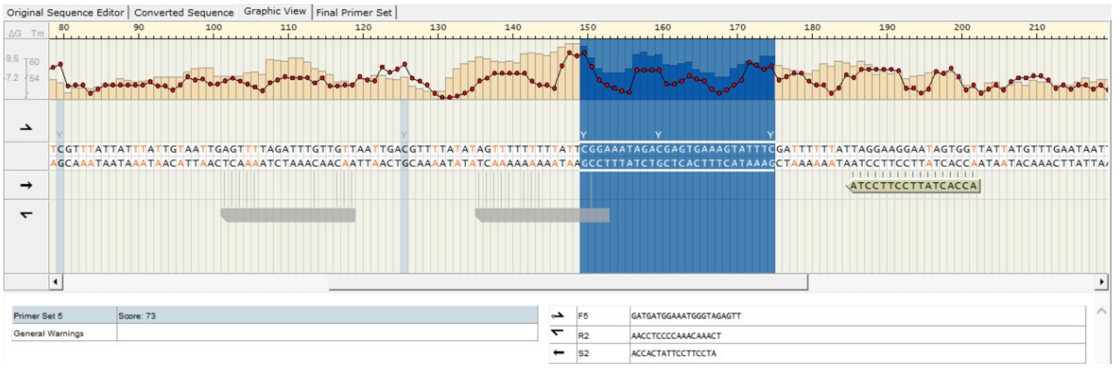

Figure S1. Pyrosequencing primer sequences in the *DLGAP2* gene. This figure shows the sequence of primer of *DLGAP2* gene in the dark area.

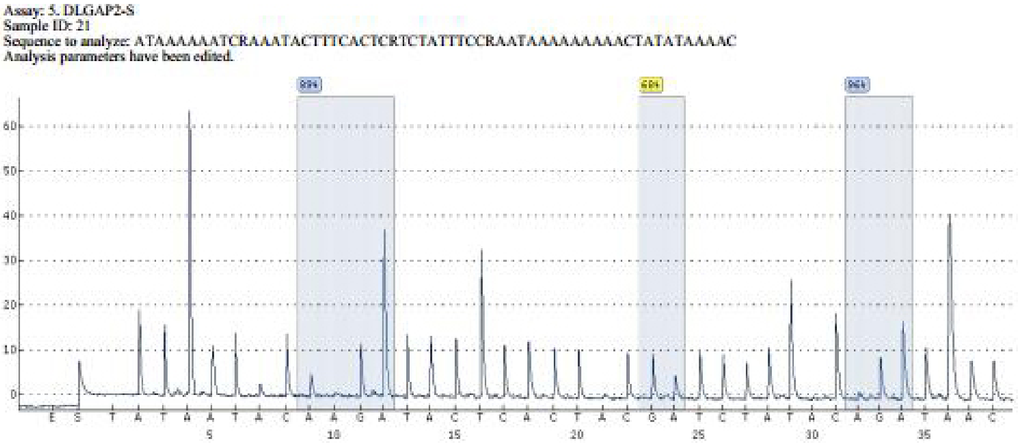

Figure S2. Peaks of three CpG sites (dark areas) in *DLGAP2* promotor region.
